# Network science approach elucidates integrative genomic-metabolomic signature of antidepressant response and lifetime history of attempted suicide in adults with major depressive disorder

**DOI:** 10.3389/fphar.2022.984383

**Published:** 2022-10-03

**Authors:** Caroline W. Grant, Angelina R. Wilton, Rima Kaddurah-Daouk, Michelle Skime, Joanna Biernacka, Taryn Mayes, Thomas Carmody, Liewei Wang, Konstantinos Lazaridis, Richard Weinshilboum, William V. Bobo, Madhukar H. Trivedi, Paul E. Croarkin, Arjun P. Athreya

**Affiliations:** ^1^ Department of Molecular Pharmacology and Experimental Therapeutics, Mayo Clinic, Rochester, MN, United States; ^2^ Department of Molecular and Integrative Physiology, University of Illinois at Urbana-Champaign, Urbana, IL, United States; ^3^ Department of Psychiatry and Behavioral Sciences, Department of Medicine, Duke Institute for Brain Sciences, Duke University, Durham, NC, United States; ^4^ Department of Psychiatry and Psychology, Mayo Clinic, Rochester, MN, United States; ^5^ Department of Quantitative Health Sciences, Mayo Clinic, Rochester, MN, United States; ^6^ Peter O’Donnell Jr. Brain Institute and the Department of Psychiatry at the University of Texas Southwestern Medical Center, Dallas, TX, United States; ^7^ Department Population and Data Sciences, University of Texas Southwestern Medical Center, Dallas, TX, United States; ^8^ Department of Internal Medicine, Division of Gastroenterology and Hepatology, Mayo Clinic, Rochester, MN, United States; ^9^ Department of Psychiatry and Psychology, Mayo Clinic, Jacksonville, FL, United States

**Keywords:** machine learning, suicide, depression, genomics, circadian rhythm, multi-omics

## Abstract

**Background:** Individuals with major depressive disorder (MDD) and a lifetime history of attempted suicide demonstrate lower antidepressant response rates than those without a prior suicide attempt. Identifying biomarkers of antidepressant response and lifetime history of attempted suicide may help augment pharmacotherapy selection and improve the objectivity of suicide risk assessments. Towards this goal, this study sought to use network science approaches to establish a multi-omics (genomic and metabolomic) signature of antidepressant response and lifetime history of attempted suicide in adults with MDD.

**Methods:** Single nucleotide variants (SNVs) which associated with suicide attempt(s) in the literature were identified and then integrated with a) p180-assayed metabolites collected prior to antidepressant pharmacotherapy and b) a binary measure of antidepressant response at 8 weeks of treatment using penalized regression-based networks in 245 ‘Pharmacogenomics Research Network Antidepressant Medication Study (PGRN-AMPS)’ and 103 ‘Combining Medications to Enhance Depression Outcomes (CO-MED)’ patients with major depressive disorder. This approach enabled characterization and comparison of biological profiles and associated antidepressant treatment outcomes of those with (*N* = 46) and without (*N* = 302) a self-reported lifetime history of suicide attempt.

**Results:** 351 SNVs were associated with suicide attempt(s) in the literature. Intronic SNVs in the circadian genes *CLOCK* and *ARNTL* (encoding the CLOCK:BMAL1 heterodimer) were amongst the top network analysis features to differentiate patients with and without a prior suicide attempt. *CLOCK* and *ARNTL* differed in their correlations with plasma phosphatidylcholines, kynurenine, amino acids, and carnitines between groups. *CLOCK* and *ARNTL*-associated phosphatidylcholines showed a positive correlation with antidepressant response in individuals without a prior suicide attempt which was not observed in the group with a prior suicide attempt.

**Conclusion:** Results provide evidence for a disturbance between CLOCK:BMAL1 circadian processes and circulating phosphatidylcholines, kynurenine, amino acids, and carnitines in individuals with MDD who have attempted suicide. This disturbance may provide mechanistic insights for differential antidepressant pharmacotherapy outcomes between patients with MDD with versus without a lifetime history of attempted suicide. Future investigations of CLOCK:BMAL1 metabolic regulation in the context of suicide attempts may help move towards biologically-augmented pharmacotherapy selection and stratification of suicide risk for subgroups of patients with MDD and a lifetime history of attempted suicide.

## Introduction

Annually, 700,000 people die by suicide worldwide, and there are 10–20 times as many non-fatal suicide attempts (Hannah et al., 2015; WHO, 2021). Patients with major depressive disorder (MDD) and prior suicide attempt(s) demonstrate lower antidepressant remission and response rates and increased antidepressant intolerance compared to those with no history of suicide attempt (Perroud et al., 2010; Kim et al., 2011). Furthermore, evaluating suicidal patients is challenging, as standard clinical risk assessments are inherently subjective (Isometsa et al., 1995; Mann et al., 2006; Smith et al., 2013). Quantitative biomarkers that identify individuals at risk for suicide attempts would assist in personalized assessment and monitoring to a) move towards pharmacotherapy individualization for subgroups of patients with MDD and a history of suicide attempt, and b) hasten referrals to suicide prevention programs.

Biological risk factors for suicide attempts include alterations in stress-response, neuronal plasticity, serotonergic signaling, lipids, and inflammatory markers such as C-reactive protein (CRP) (De Berardis et al., 2008; Oquendo et al., 2014; Sudol and Mann, 2017; De Berardis et al., 2020). However, there are currently no biomarkers (laboratory or imaging) that are sufficiently valid for routine clinical stratification of suicide risk (Ryan and Oquendo, 2020). Enhanced understanding of the interplay between biomarkers for lifetime suicide attempt(s) and biomarkers for outcomes to antidepressant pharmacotherapy represents a first step towards personalized MDD care for individuals with a lifetime history of suicide attempts.

**FIGURE 1:**
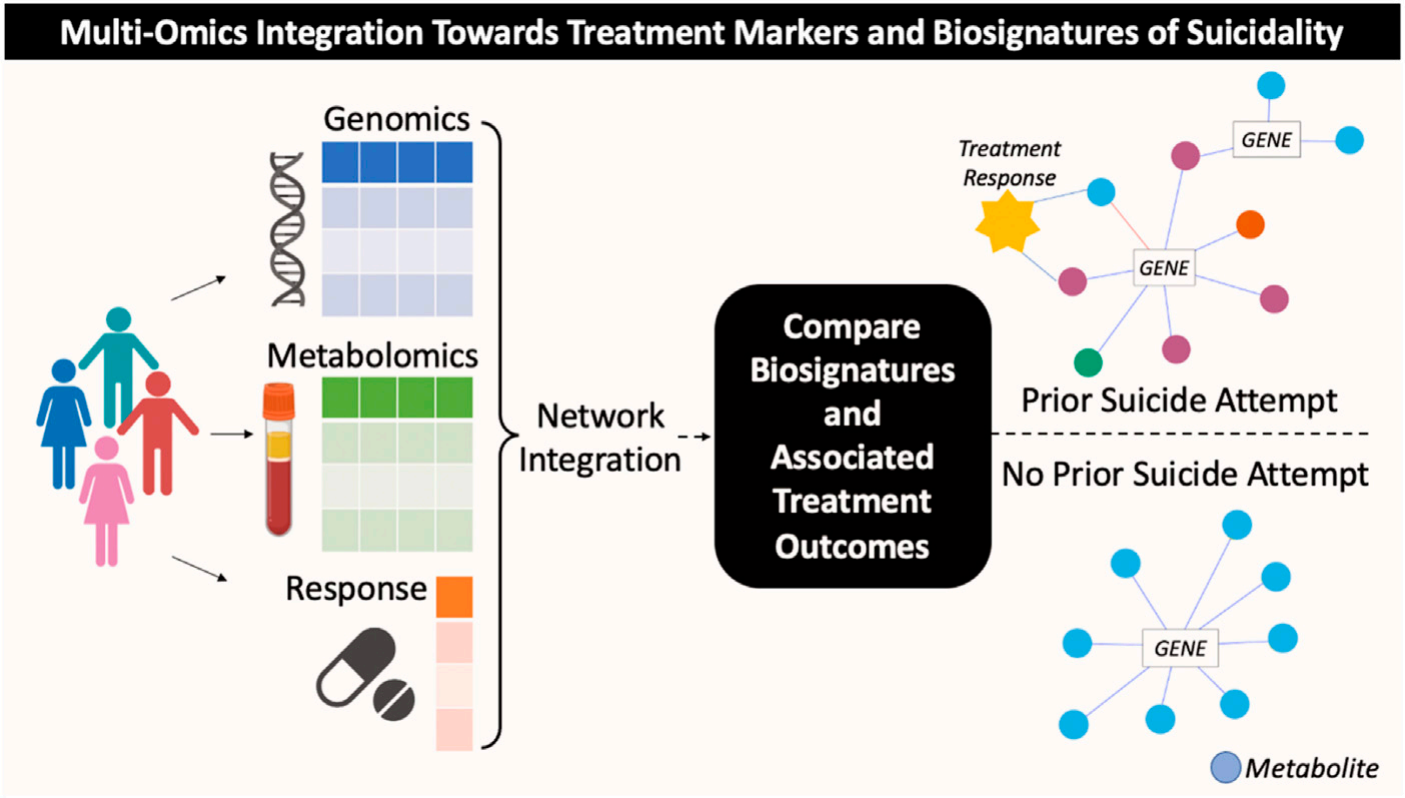
Conceptual overview of multi-omics integration analysis towards identification of candidate treatment markers for subgroups of patients with MDD based on history of suicide attempt.

The biological factors which contribute to a) suicide risk and b) antidepressant treatment outcomes likely comprise multiple pathological processes interacting together as a network (Kouter and Videtic Paska, 2021). Integrative omics methods (i.e., genomics, metabolomics, transcriptomics, epigenomics), therefore, are novel and promising approaches for elucidating important molecular mechanisms underlying suicidality and other complex behavioral phenotypes (Barabasi et al., 2011; Bhak et al., 2019; Joyce et al., 2021; Liu et al., 2021; Grant et al., 2022). Integrating genomics and metabolomics has been particularly informative for identifying novel biomarkers of MDD risk and antidepressant response (Gupta et al., 2016; Athreya et al., 2018; Liu et al., 2018; Athreya et al., 2019; Liu et al., 2020). Together, the genome and metabolome capture complimentary information of heritable and environmentally driven suicide attempt risk factors. The heritability of suicide attempts is high, estimated at 17%–55% (Mullins et al., 2022), and the metabolome shows long-term alterations following traumatic events, (e.g., suicide attempts) (Bernini et al., 2009; Ghini et al., 2015). The current study, therefore, used a partial-least squares regression-based network science approach to integrate genomics, metabolomics, lifetime suicide attempt history, and treatment outcomes to a) understand if genetically driven metabolomic differences exist in individuals with a lifetime history of attempted suicide, and b) assess if such biological differences may have potential implications for antidepressant treatment outcomes (Figure 1 for conceptual overview).

This work hypothesized that biological signatures for suicide attempters within a sample of patients with MDD could be developed by integrating metabolomics and genomics data. Integration of -omic data with treatment outcomes demonstrates that the top metabolomic and genomic features to differentiate MDD patients with and without a lifetime history of attempted suicide correlate with antidepressant treatment response. The findings of this work would generate implications for potential therapeutic utility for the biomarkers which distinguish patients with MDD with a lifetime history of suicide attempts.

## Materials and methods

### Data sources

This was a cross-sectional secondary analysis of clinical and blood biomarker data from adults with MDD who participated in the Pharmacogenomics Research Network Antidepressant Medication Study (PGRN-AMPS) and Combining Medications to Enhance Depression Outcomes (CO-MED) studies. Subjects were included if they had metabolomics, genomics, and a binary clinical measure (yes/no) of prior suicide attempt (Table 1; see Supplementary Figure S1 for sample inclusion). Both the PGRN-AMPS and CO-MED trials have been previously described (Rush et al., 2011; Ji et al., 2013). Briefly, the Mayo Clinic PGRN-AMPS trial (NCT00613470) enrolled 927 outpatients with nonpsychotic MDD who scored ≥14 on the 17-item Hamilton Depression Rating Scale (HAMD-17 (Hamilton, 1967)) at enrollment; of these 245 participants had metabolomics, genomics, and a self-reported lifetime history of at least one attempted suicide. The CO-MED trial (NCT00590863), conducted at 15 sites throughout the United States, enrolled 665 outpatients who met DSM-IV-TR criteria for recurrent or chronic (current episode lasting ≥2 years) nonpsychotic MDD and scored ≥16 on the HAMD-17 at study entry; of these 103 participants had metabolomics, genomics, and a self-reported lifetime history of at least one attempted suicide. Thus, for these analyses, a total of 348 participants were included. All PGRN-AMPS and CO-MED participants provided written informed consent and each study was approved by their respective Institutional Review Boards.

**TABLE 1:** Demographics and clinical characteristics.

	History of suicide attempt (N = 46)	No history of suicide attempt (N = 302)	*p*-value
Study Number [PGRN-AMPS, CO-MED]	38, 8	207, 95	0.057
Sex [% Female]	74%	65%	0.31
Age [mean (SD)]	37.9 (12.5)	42.0 (13.0)	0.085
Years of education [mean (SD)]	14.5 (2.7)	14.6 (2.5)	0.60
Race [% White]	93%	92%	1
Race [% Black or African American]	5%	5%	1
Race [% Other]	2%	3%	1
Ethnicity [% Hispanic]	17%	13%	0.53^a^
Depression onset < age 18 [%]	65%	37%	0.00060*
Total QIDS-C at enrollment [mean (SD)]	16.5 (3.4)	14.9 (3.3)	0.0027*
QIDS-C sleep-onset insomnia [mean (SD)]	1.9 (1.2)	1.5 (1.3)	0.066
QIDS-C mid-nocturnal insomnia [mean (SD)]	1.9 (1.1)	2.0 (1.1)	0.82
QIDS-C early morning insomnia [mean (SD)]	0.6 (1.0)	0.9 (1.2)	0.067
QIDS-C hypersomnia [mean (SD)]	0.7 (1.1)	0.7 (1.0)	0.37
QIDS-C energy/fatiguability [mean (SD)]	1.7 (0.8)	1.7 (0.7)	0.70
QIDS-C Antidepressant Response Rates [%]	63.0%	68.5%	0.57
Citalopram [%]	39.1%	26.8%	0.11
Escitalopram [%]	47.7%	51.7%	0.87
Escitalopram/Bupropion [%]	4.3%	10.2%	0.28
Venlafaxine/Mirtazapine [%]	8.7%	11.3%	0.80

*Significantly different (*p* ≤ 0.05) by *t*-test (continuous variables), Fisher’s Exact test (categorical variables), or Kruskal–Wallis test (ordinal variables).

a Ethnicity characterizations are based on 38 individuals with a history of suicide attempt and 256 individuals without a history of suicide attempt according to data availability. QIDS-C, sleep items are collected as: 0 = Absent, 1 = Less than half the time, 2 = More than half the time, 3 = Almost all the time. Items for the energy/fatigue and sleep disturbance domains are reported given the relevance for circadian rhythms. For all items, see supplementary Table SIV.

### Measures and outcomes

Self-reported lifetime history of suicide attempts (yes/no) was collected via a baseline case report form in PGRN-AMPS (“Has patient ever attempted suicide?“) and *via* the SAD PERSONS scale (Patterson et al., 1983) in CO-MED. Depression severity was assessed with the 16-item Quick Inventory of Depressive Symptomatology—Clinician-Rated (QIDS-C) (Trivedi et al., 2004). The QIDS-C tallies responses to 16 questions to derive a total score that ranges from 0 (no depression) to 27 (very severe depression). These 16 questions cover the nine diagnostic and statistical manual (DSM)-IV MDD symptom criterion domains: 1) sad mood; 2) concentration; 3) self-criticism; 4) suicidal ideation; 5) interest; 6) energy/fatigue; 7) sleep disturbance (initial, middle, and late insomnia or hypersomnia); 8) decrease/increase in appetite/weight; and 9) psychomotor agitation/retardation. Participants were classified as responders or non-responders at 8 weeks after initiating treatment with citalopram, escitalopram, bupropion plus citalopram, or venlafaxine plus mirtazapine. Responders were defined as demonstrating ≥50% reduction in total QIDS-C score at (Table 1).

### Genomics

PGRN-AMPS patients were genotyped with Illumina human 610-Quad BeadChips (Illumina, San Diego, CA, United States), with quality control and imputation as previously reported (Gupta et al., 2016). CO-MED patients were genotyped, as previously published, with Illumina Quad, Human Omni 2.5 bead chips (Gadad et al., 2018). Imputation was performed using the Michigan Imputation Server (Das et al., 2016). All CO-MED single nucleotide variants (SNVs) included in this analysis met imputation *R* ^2^ and call rates of >0.95, while genotyped SNVs met LooRsq >0.95.

### Candidate single nucleotide variant search strategy

**FIGURE 2:**
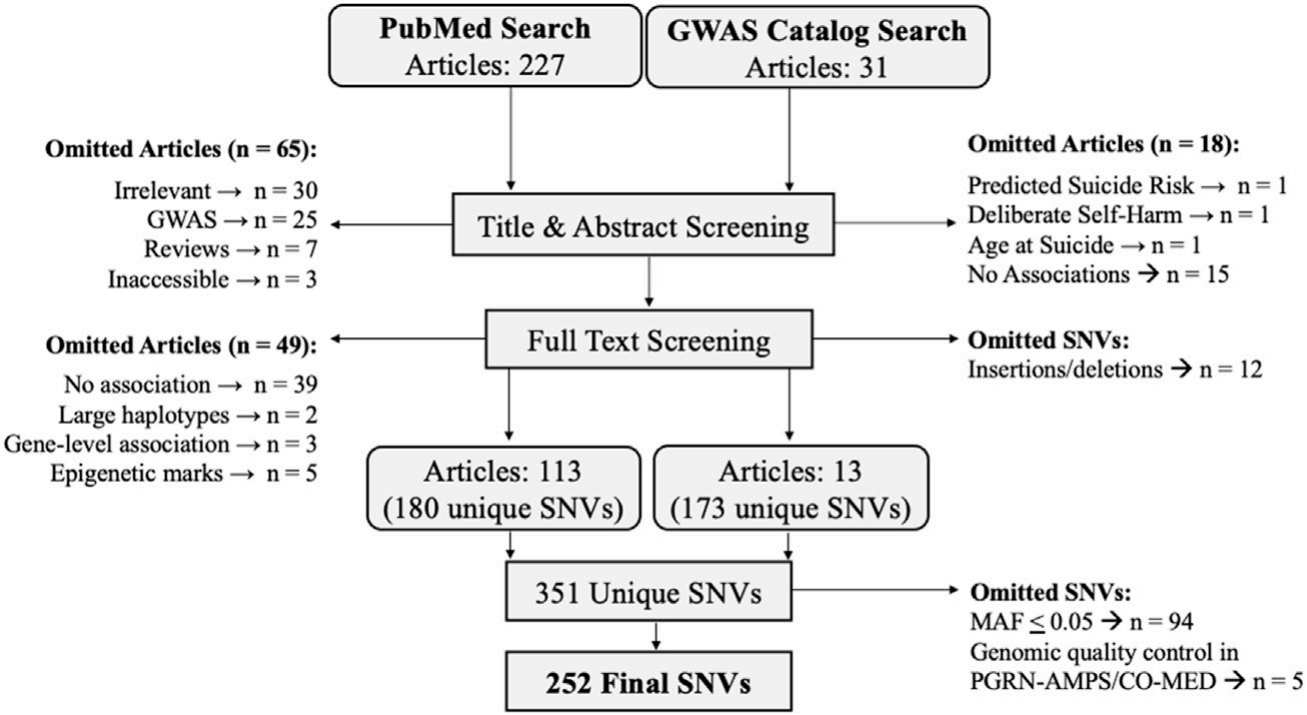
Single nucleotide variation (SNV) search strategy.

Candidate SNVs that best differentiated individuals with and without a prior suicide attempt were identified by a PubMed Search and a GWAS Catalog (Buniello et al., 2019) Search (Illustrated in Figure 2). These complimentary strategies enabled the identification of SNVs which associated with suicide attempts in both targeted (e.g., candidate gene) and unbiased (e.g., genome-wide association study (GWAS)) studies. These searches were conducted agnostic of clinical diagnoses, as the heritability of suicidal behavior is transdiagnostic (Oquendo et al., 2014; Mann and Rizk, 2020). The PubMed Search, conducted on 07/09/2021, included the following criteria: ((SNP) OR (SNPs) OR (single nucleotide polymorphism) OR (single nucleotide polymorphisms) OR (SNV) OR (SNVs) OR (single nucleotide variant) OR (single nucleotide variants)) AND ((suicide attempt) OR (suicide attempts)). SNVs which reached significance according to their respective study definitions were retained for evaluation in the PGRN-AMPS and CO-MED cohorts. The GWAS Catalog search was conducted on 07/20/2021 using the gwasrappid R package (Magno and Maia, 2020), which enabled efficient querying and downloading of SNVs associated with suicide attempts. GWAS Catalog SNVs meeting the catalogue’s definition of suggestive significance (*p* < 1.0 × 10^−5^) were retained for further evaluation. Studies were reviewed by two independent raters. Studies and SNVs were excluded based upon criteria outlined in Figure 2. SNVs were annotated with genomic location and nearest gene according to the National Center for Biotechnology Information (NCBI) Reference Sequence Database (RefSeq) using haploR (Zhbannikov et al., 2017), implemented in R 4.0.3 (R Foundation for Statistical Computing, 2020) using RStudio version 1.3 (RStudio, 2020).

### Single nucleotide variant associations with suicide attempt history

Candidate SNVs meeting the quality control criteria (outlined in the **Genomics** section) and a minor allele frequency threshold of 5% were assessed for association with lifetime history of attempted suicide in the combined PGRN-AMPS and CO-MED cohorts using chi-squared tests with two degrees of freedom. As this was intended as a discovery step to identify SNVs which may contribute to multi-omics differentiation of patients by lifetime history of suicide attempt, a nominal *p*-value of ≤0.05 was considered significant.

### Metabolomics

In both the PGRN-AMPS and CO-MED cohorts, plasma metabolites were assayed with the AbsoluteIDQ p180 platform (BIOCRATES Life Science AG, Innsbruck, Austria) (Sciences, 2010). This targeted assay detects metabolites from five analyte classes (acylcarnitines, amino acids, biogenic amines, glycerophospholipids and sphingolipids) by triple quadrupole tandem mass spectrometry operated in Multiple Reaction Monitoring mode. Metabolomic profiling and quality control were conducted as previously published (Czysz et al., 2019; Ahmed et al., 2020; MahmoudianDehkordi et al., 2021), and metabolites with ≥10% missingness were excluded. 153 metabolites met quality control criteria in both the PGRN-AMPS and CO-MED datasets (Supplementary Table S1). The p180 platform has demonstrated utility in characterizing related psychiatric phenotypes, including MDD and antidepressant response (Gupta et al., 2016; Neavin et al., 2016; Athreya et al., 2018; Bhattacharyya et al., 2019; Czysz et al., 2019; Ahmed et al., 2020; Joyce et al., 2021; MahmoudianDehkordi et al., 2021; Brydges et al., 2022), schizophrenia (Parksepp et al., 2020), and psychosis (Kriisa et al., 2017; Balotsev et al., 2019), but has not yet been investigated in the context of suicidality. Therefore, all metabolites meeting quality control criteria were included in the current integration analysis. This enabled the assessment of promising analyte classes in the characterization of suicide attempt, with the limitation that the p180 platform does not capture other metabolites of potential etiologic significance for neuropsychiatric disorders or drug response [i.e., dopamine, γ-aminobutyric acid (GABA)].

### Network science for the integration of multi-omics and antidepressant outcomes

Integrative network analysis was performed to a) characterize the multi-omics profiles of patients with and without a lifetime history of suicide attempt, b) identify SNVs, metabolites, and associated treatment outcomes which best differentiate the profiles of patients with and without a lifetime history of suicide attempt, and c) detect sub-networks (i.e., communities) of tightly correlated SNVs, metabolites, and treatment outcomes to elucidate novel biological associations which may reflect biological mechanisms of attempted suicide. Subnetworks are hypothesized to consist of functionally related biomolecules (Barabasi et al., 2011; Lichtblau et al., 2017).

This network science approach, based on partial least squares regression, represents a multi-variate dimensionality reduction tool that is becoming increasingly popular in integrative omics analyses (Ruiz-Perez et al., 2020). Input features included: a) SNVs associated with prior suicide attempt in the PGRN-AMPS and CO-MED cohorts (*p* ≤ 0.05), b) all 153 metabolites meeting quality control criteria, and c) treatment response measured at 8 weeks. The resulting networks comprise SNVs, metabolites, and associated outcomes which are significantly correlated (|r|>0.1, *p* < 0.05) in patients with and without a lifetime history of suicide attempt. The derived networks were compared by calculating the delta centrality (change in eigenvector centrality) for each SNV and metabolite across networks. Delta centrality ranges from 0 to 1, with 1 indicating significant differences between patients with and without a history of suicide attempt in the number and quality of network correlations (Lichtblau et al., 2017).

Prior to integration analysis, the sole missing datapoint (one taurine value) was imputed with K-nearest neighbor’s imputation. Metabolites were transformed using Yeo-Johnson transformation, centered at zero, and scaled to unit variance. Preprocessing was performed using the tidymodels package version 0.1.2 (Tidymodels, 2020) and integration analysis was performed using xMWAS (Uppal et al., 2018), implemented in R 4.0.3^38^ using RStudio version 1.3^39^.

### Resource availability

The PGRN-AMPS data has been deposited in dbGaP (study accession phs000670. v1. p.1). Funding for the CO-MED project was exclusively provided through institutional resources. Data supporting the conclusions of this article will be made available by contacting the authors, without undue reservation.

## Results

### Participant characteristics

Participants were predominantly female (66%) and Caucasian (92%). Of the 348 participants included, 46 (13%) had a lifetime history of a suicide attempt. Individuals who reported a prior suicide attempt were more likely to have a depression onset prior to age 18 (65% vs. 37%; *p* < 0.05) and had higher QIDS-C scores (16.5 vs.14.9) compared with individuals without a lifetime history of attempted suicide. Table 1 provides the demographics and clinical characteristics of the sample.

### Candidate single nucleotide variant search

The PubMed Search for SNVs associated suicide attempts yielded 227 articles, and 113 were retained following title, abstract, and full text screening. The GWAS Catalog Search yielded 31 articles, 13 of which were retained following identical screening (Figure 2). In total, 351 unique SNVs were associated with suicide attempts in a transdiagnostic manner in the literature, with some replicating in multiple studies (for a complete table of SNVs, SNV annotations, and studies see Supplementary Table S2). SNVs which associated with suicide attempt(s) in the GWAS catalog most frequently localized outside the open reading frame (N = 110 SNVs), while SNVs derived from the PubMed search most frequently localized to intronic genomic regions (N = 125 SNVs) (Table 2). The GWAS catalog studies most frequently reported associations of RUNX1 with suicide attempt(s), while the PubMed studies most frequently reported associations with FKBP5 (Table 2). The initial literature associations of these SNVs and suicide attempts were discovered in cohorts with a variety of psychiatric diagnoses: bipolar disorders, substance dependence, childhood onset mood disorders, MDD, eating disorders, schizophrenia, post-traumatic stress disorder, mixed cohorts, and no specified diagnosis (Table 2).

**TABLE 2:** GWAS catalog and PubMed search findings.

GWAS catalog search		PubMed search	
SNV Annotations			
Outside the open reading frame	110	Intronic variant	125
Intronic Variant	60	Outside the open reading frame	31
Synonymous Variant	2	Non-synonymous variant	22
Untranslated 3′ Region	1	Untranslated 3′ Region	22
		Synonymous Variant	21
Most Frequently Studied Genes (N studies > 5)			
*RUNX1*	8	*FKBP5*	15
*NAV3*	7	*CRHR1*	12
		*HTR2A*	12
		*COMT*	10
		*BDNF*	8
		*ABCB1*	7
		*CCKBR*	7
		*TPH2*	7
		*SCN8A*	6
Cohorts in which SNVs associated with suicide attempt(s)			
Bipolar Disorder, MDD, or Schizophrenia	79	Mixed psychiatric cohort, or no diagnosis	80
Mixed Psychiatric Cohort, or No Diagnosis	59	Bipolar Disorder	42
PTSD, MDD, or Neither	21	MDD	36
Bipolar Disorder	7	Mood Disorder	22
Bipolar I and II or MDD	7	Schizophrenia	18
		Alcohol, Cocaine, or Opioid Dependence	9
		Substance Dependence	7
		Alcohol Dependence	4
		Disruptiveness (children)	1
		Eating Disorders	1
		Schizophrenia or Schizoaffective Disorder	1

SNV, annotations from NCBI’s Single Nucleotide Polymorphism Database (dbSNP), with number of SNVs, reported for each category. Most frequently studied genes are reported as the number of SNVs, mapping to each gene (by distance, according to the NCBI, RefSeq Database) with >5 studies. PTSD: post-traumatic stress disorder.

### Single nucleotide variant associations with suicide attempt history

Genotypes for 252 of the 351 candidate SNVs were obtained from PGRN-AMPS and CO-MED patients after quality control and applying a minor allele frequency threshold of 5% (Figure 2 for SNV exclusion). Chi-square tests were performed to associate SNVs with suicide attempt history in these depressed adults, with twelve SNVs meeting nominal significance (*p* ≤ 0.05) (Supplementary Table S3 for complete results). After pruning for linkage disequilibrium, eight significant SNVs from distinct genomic regions remained (Table 3). The initial literature associations of these SNVs and suicide attempts were discovered in cohorts with a variety of psychiatric diagnoses (Table 3) (Fudalej et al., 2010; Roy et al., 2010; Strauss et al., 2012; Pawlak et al., 2015; Penas-Lledo et al., 2015; Acikel et al., 2020; Russell et al., 2020).

**TABLE 3:** SNVs associated with history of suicide attempt.

SNV	Location	Gene	SNV annotation	MAF	χ2	p	Discovery cohort
rs1982350	11:13328584	*ARNTL*	Intronic	0.64	18.85	8.1E-5	Bipolar disorder
rs1045642	7:87509329	*ABCB1*	Synonymous	0.47	13.11	0.0014	N/A
rs3777747	6:35611225	*FKBP5*	Intronic	0.46	8.27	0.016	Substance dependence
rs1386483	12:72018714	*TPH2*	Intronic	0.58	7.40	0.025	N/A
rs11105336	12:77470960	*NAV3*	5′ 360327bp	0.38	7.00	0.030	N/A
rs705315	7:98629424	*NPTX2*	3′ UTR	0.06	6.71	0.035	Childhood onset mood disorders
rs3805148	4:55440643	*CLOCK*	Intronic	0.35	6.65	0.036	Bipolar disorder
rs1171276	1:65521766	*LEPR*	Intronic	0.15	6.52	0.038	Depressed adolescents

SNVs which are significantly different (*p* ≤ 0.05) by chi-squared tests with two degrees of freedom between individuals with and without a history of suicide attempt. Gene annotations and locations are according to the NCBI, Reference Sequence Database (RefSeq) and dbSNP, pulled using the haploR package. Diagnosis indicates the cohort of patients in which this variant associated with suicidality in our literature and GWAS, catalog variant search. N/A: no specified diagnosis. UTR, Untranslated Region. bp, base-pairs. Location, chromosome:position. MAF, Minor allele frequency. Gene names are official gene symbols provided by the HUGO gene nomenclature committee (HGNC).

### Network science for the integration of multi-omics and antidepressant outcomes

**FIGURE 3:**
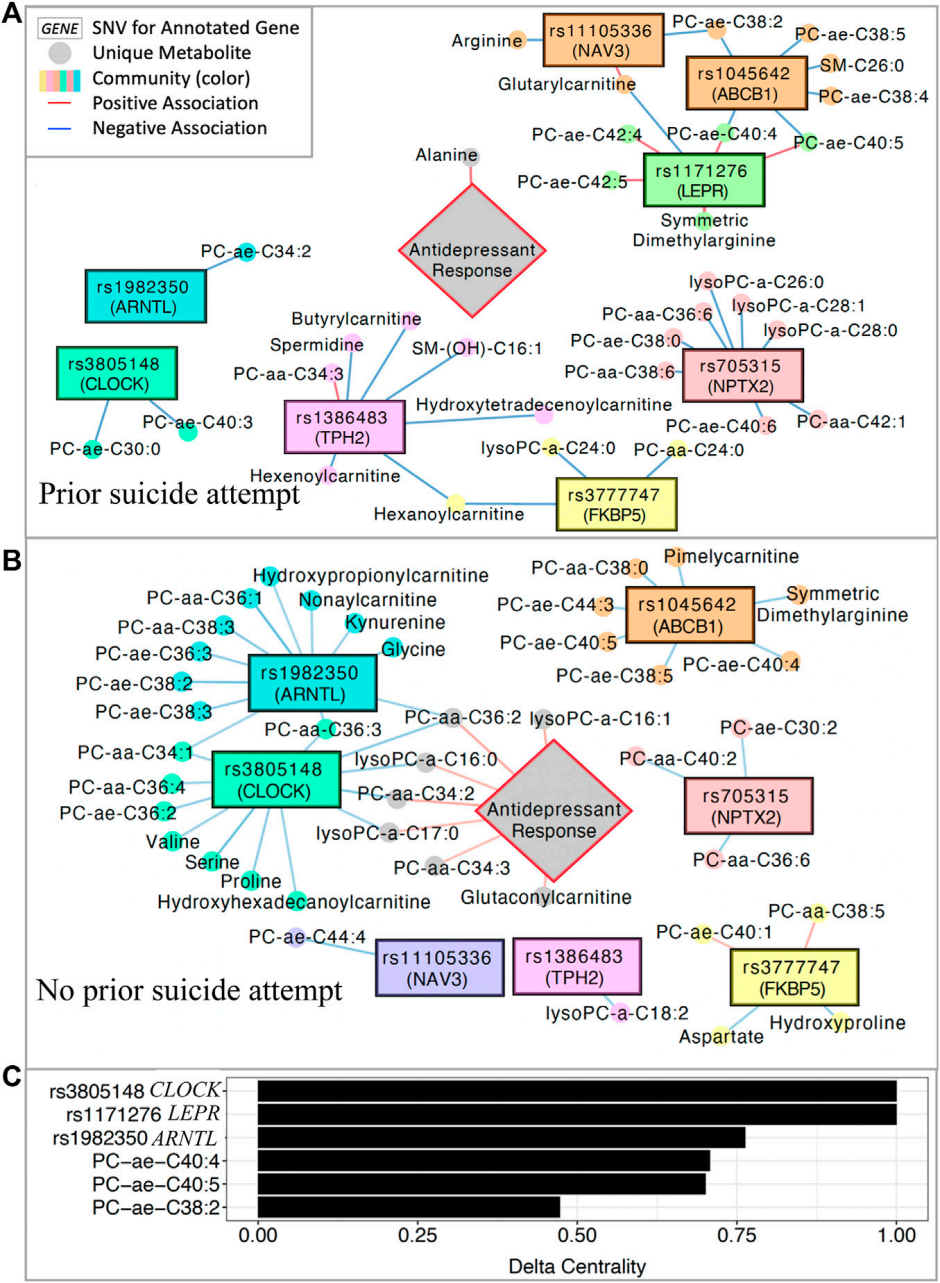
Multi-omics integration network. **(A)** Significantly associated SNVs and metabolites in patients with a history of suicide attempt and **(B)** patients with no history of suicide attempt. **(C)** Delta centrality scores for the five variables with the highest delta centrality values. Delta centrality is defined as the change in eigenvector centrality across networks (lifetime history of suicide attempt vs. no history). Delta centrality ranges from 0–1, with 1 indicating the greatest differences in number and quality of connections across networks. PC: phosphatidylcholine; SM: sphingomyelin.

Integrative network analysis was performed to characterize and compare the multi-omics profiles of patients with (Figure 3A) and without (Figure 3B) a lifetime history of attempted suicide. The analysis integrates measures and detects subnetworks of biological and clinical features (e.g., SNVs, metabolites, treatment outcomes) which are highly correlated within their subnetwork, but sparsely correlated to the rest of the network (Girvan and Newman, 2002; Uppal et al., 2018).

After inputting all 153 metabolites, the eight significant SNVs, and treatment outcomes into xMWAS (Uppal et al., 2018), eight subnetworks of correlated SNVs and metabolites were detected in patients with and without a lifetime history of attempted suicide (Figures 3A,B). Delta centrality was calculated for each feature, with higher values representing larger differences across networks (history suicide attempt vs. no history of suicide attempt) in the strength and quality of network connections. The six SNVs and metabolites with the highest delta centrality are shown in Figure 3C. These included three intronic SNVs with high (>0.75) delta centrality in *CLOCK*, *LEPR*, and *ARNTL* (rs3805148, rs1171276, rs1982350, respectively).

In patients with a previous suicide attempt, the *ARNTL* and *CLOCK* SNVs were significantly correlated (*p* < 0.05) with only one and two phosphatidylcholines, respectively (Figure 3A). In such patients, antidepressant response correlated positively with alanine concentrations. Additionally, the *LEPR* SNV was positively correlated with long-chain phosphatidylcholines, symmetric dimethylarginine, and glutarylcarnitine in patients with a history of suicide attempt.

In contrast, in patients with no lifetime history of attempted suicide, the *ARNTL* and *CLOCK* SNVs shared significant negative correlations (*p* < 0.05) with three phosphatidylcholine metabolites (Figure 3B). Individually, the *ARTNL* and *CLOCK* SNVs were also negatively correlated (*p* < 0.05) with many additional phosphatidylcholines, kynurenine, amino acids (serine, proline, valine, glycine), and carnitines. Four phosphatidylcholine metabolites associated with *ARNTL* and *CLOCK* SNVs are positively correlated with treatment response in individuals with no prior suicide attempt. The *LEPR* SNV was not correlated with any metabolites in patients without a history of suicide attempt. Pearson correlation coefficients for all SNVs and metabolites represented in these networks are tabulated in Supplementary Table S4.

## Discussion

This work using network science approaches identified integrative genomic-metabolomic signatures of antidepressant response and lifetime history of attempted suicide in adults with MDD. Importantly, the subnetworks of biomeasures which most differentiated individuals by their lifetime history of suicide attempt (*CLOCK*, *ARNTL* subnetworks) correlated with response to antidepressant pharmacotherapies exclusively in individuals without a prior suicide attempt. Future investigations of CLOCK:BMAL1 metabolic regulation in the context of suicide attempts may help move towards biologically-augmented pharmacotherapy selection and stratification of suicide risk and for patients with MDD based upon their lifetime history of attempted suicide.

The network science approach utilized in this work is a multivariate analysis that provides an analytical prioritization of biomarker subnetworks for future mechanistic investigation (Barabasi et al., 2011; Joyce et al., 2021). SNVs in the circadian genes *CLOCK* and *ARNTL* and their associated plasma metabolites discriminate individuals with and without a lifetime history of attempted suicide, despite all patients having the same diagnosis of MDD. Circadian genes including *CLOCK* and *ARNTL* have been implicated in suicidality previously, yet the underlying molecular mechanisms, including the downstream effects on metabolism, and the treatment implications are incompletely characterized (Benedetti et al., 2015; Levey et al., 2016).

**FIGURE 4:**
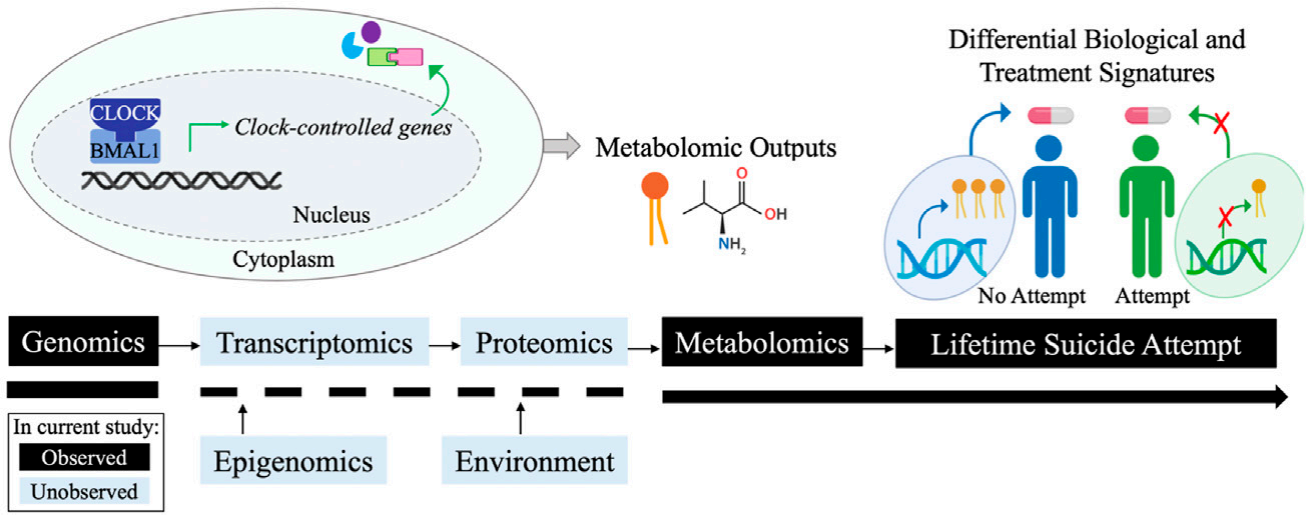
Conceptual summary. Simplified circadian rhythm loop. Tuning of such loops occurs by multiple mechanisms (blue boxes). Black boxes denote available data and blue boxes denote unavailable data in the current analysis. Figure created with BioRender.com.

The *CLOCK* and *ARNTL* genes encode for the CLOCK (circadian locomotor output cycles kaput) and BMAL1 (brain and muscle aryl hydrocarbon nuclear translocator (ARNT)-like 1) proteins, respectively. These are core components of the molecular circadian mechanism, which comprises the autonomous clocks of nearly every cell in the body and the “master circadian pacemaker” of the hypothalamic suprachiasmatic nucleus (Mohawk et al., 2012; Rijo-Ferreira and Takahashi, 2019). The sleep/wake cycle is perhaps the most evident function of circadian rhythms, but many additional physiological processes are regulated by circadian rhythms, including feeding, temperature, secretion of hormones, metabolism of drugs and xenobiotics, glucose homeostasis, and cell cycle progression (Rijo-Ferreira and Takahashi, 2019). The associations derived in this work suggest underlying biological differences between patients with and without a prior suicide attempt in the mechanistic processes which connect circadian rhythm variation (e.g., SNVs in *CLOCK*, *ARNTL*) with downstream associated metabolites (e.g., phosphatidylcholines, kynurenine, and more). Altered transcription regulation may represent one such mechanism, as these SNVs are expression quantitative trait loci (eQTLs) which alter messenger RNA (mRNA) levels (*p* < 0.001) of the *ARNTL* and *CLOCK* transcripts in multiple peripheral tissues according to the Genotype-Tissue Expression (GTEx) database (Consortium, 2020). While genomic, metabolomic, and clinical data was available for analysis in this study, the epigenomic, transcriptomic, proteomic, and environmental factors which may mediate the derived genomic and metabolomic associations must be examined in future work to mechanistically understand these correlations (Figure 4).

Phosphatidylcholines were network correlates of *ARNTL* and *CLOCK* SNVs that differed between individuals with and without a lifetime history of attempted suicide. Phosphatidylcholines are a subclass of phospholipid and the main component of mammalian membranes and lipoproteins (van der Veen et al., 2017). Phosphatidylcholines are implicated in MDD risk (Liu et al., 2015; Knowles et al., 2017; Walther et al., 2018; Pu et al., 2020) and response to antidepressant treatments (Joyce et al., 2021; MahmoudianDehkordi et al., 2021), but the relationship between suicidality and phosphatidylcholines is yet to be understood. However, functionally related lipids, including cholesterol, have been implicated in suicidal behavior (Muldoon et al., 1990; Neaton et al., 1992; Salter, 1992; Morgan et al., 1993; Maes et al., 1997; Yang et al., 2003; Alghamdi et al., 2018). In some trials, the benefits of statins, which lower cholesterol in individuals with high lipid levels, have been offset by increased suicidality (Knowles et al., 2017). These findings encourage additional evaluation of the lipidome, including phosphatidylcholines, in suicidality and the risk of attempted suicide.

Kynurenine is a biogenic amine produced *via* tryptophan metabolism (Peters, 1991) which has been separately associated with a) circadian disturbance (e.g., sleep disruption) (Bhat et al., 2020), b) suicidal behavior and ideation (Rujescu et al., 2003; Lin and Tsai, 2004; Pitchot et al., 2005; Lopez et al., 2007; Malone et al., 2007; Yeh et al., 2014; Setoyama et al., 2016), and c) antidepressant response (Sun et al., 2020). Suicidal patients exhibit dysregulation along the tryptophan metabolism pathway at the level of enzymes (e.g., tryptophan hydroxylase I, II (TPH1, TPH2)) (Rujescu et al., 2003; Lopez et al., 2007), receptors (5-Hydroxytryptamine Receptors 5HT1A, 5-HT2A) (Pitchot et al., 2005; Malone et al., 2007), and transporters (serotonin transporter (5-HTTLPR)) (Lin and Tsai, 2004; Yeh et al., 2014). Sleep deprivation activates enzymatic degradation of tryptophan along the kynurenine pathway, resulting in decreased production of melatonin and neuroprotective metabolites (e.g., kynurenic acid) and increased production of inflammatory and neurotoxic metabolites (e.g., 3-hydroxykynurenine, quinolinic acid) (Bhat et al., 2020). The network analysis unifies these observations and identifies kynurenine as part of the *ARNTL* subnetwork of circadian SNVs associating with antidepressant response exclusively in MDD patients with a lifetime history of suicide attempt and not in those without a lifetime history of suicide attempt. This encourages future mechanistic investigation of the negative correlation between the circadian variant rs1982350 (in *ARNTL*)*,* the subnetwork’s additional metabolites (e.g., phosphatidylcholines), and kynurenine in the context of antidepressant response and lifetime history of suicide attempts.

The results of this study also extend beyond circadian regulation of metabolism to identify additional promising biomarkers of suicide attempt. Eight SNVs were included in the network analysis because they associated with suicide attempts both in various psychiatric cohorts in the literature (Fudalej et al., 2010; Roy et al., 2010; Strauss et al., 2012; Pawlak et al., 2015; Penas-Lledo et al., 2015; Acikel et al., 2020; Russell et al., 2020) and in PGRN-AMPS and CO-MED adults with MDD. These SNVs may, therefore, represent promising transdiagnostic markers of suicide attempt, and may be validated across additional psychiatric cohorts in the future. An intronic SNV in *LEPR* (leptin receptor), which is an eQTL for *LEPR* in whole blood and lymphoblastoid cells, demonstrated high network delta centrality (delta centrality = 1), showing differential associations with plasma phosphatidylcholines, symmetric dimethylarginine, and hydroxyvalerylcarnitine in patients with and without a lifetime history of attempted suicide. Enhancing our understanding of these associations via future functional experimentation may assist in pharmacogenomic personalization of therapeutics which modulate these systems. While further evidence is needed to assess whether targeting these systems through pharmacotherapy might benefit subgroups of patients with MDD, compounds exist already which can modulate these systems. For example, orexin modulators such as Orexin A, can modulate leptin receptor signaling (Medrano et al., 2018). Orexin modulators have also demonstrated antidepressant and sleep-promoting benefits in patients with major depressive disorder (Recourt et al., 2019).

This study has limitations. Lifetime history of suicide attempt was self-reported, with no independent verification. Information on the severity of the suicide attempt and the number of past such attempts was not available for examination. A larger proportion of patients with a history of suicide attempt had depression onset prior to age 18 compared to those without a history of suicide attempt 65% vs. 37%), which aligns with epidemiological reports (Claassen et al., 2007). The enrollment criteria of the PGRN-AMPS and CO-MED trials indicated that participants had, on average, a moderate to severe MDD presentation (Rush et al., 2006). To evaluate the generalizability of our findings across the spectrum of MDD severity, our study’s biomarker associations must be evaluated in future large cohorts (e.g., treatment-resistant depression, very severe depression). This study’s samples did not have a sex difference between those with and without a lifetime history of suicide attempt, despite epidemiological evidence which suggests that females more frequently attempt suicide than males (Freeman et al., 2017). This may reflect the sample’s moderate depressive severity or additional factors related to study enrollment, indicating replication of biosignatures in additional cohorts is warranted. These analyses were not controlled for age at which the suicide attempt occurred (as this information was not collected) or sex (due to limited males with a lifetime of attempted suicide in these datasets, *N* = 12). There are known clinical and demographic differences in the suicide attempts of young, middle-aged, and older individuals (e.g., reason for attempt, propensity to re-attempt, occupation, alcohol-co-ingestion, psychiatric history, sex, and hospital discharge outcomes) (Freeman et al., 2017; Kim et al., 2020), but it is unknown whether these factors might influence the derived biosignatures. Future studies should utilize cohort designs to investigate the impact of sex, age, and additional clinical/demographic factors at suicide attempt on genomic-metabolomic biosignatures of suicide attempt. This could confirm whether the current findings generalize across patients with prior suicide attempts. Such future sex-stratified analyses are especially warranted given differential expression of clock genes in adipose tissue of men and women, which may also generalize to additional tissues (e.g., brain) (Gomez-Abellan et al., 2012; Levey et al., 2016; Labonte et al., 2017). The PGRN-AMPS and CO-MED cohorts largely comprise Caucasian individuals, and the SNVs identified via the literature search vary in minor allele frequency across populations (Supplementary Table S5). These network interactions should be replicated in larger cohorts and across ancestries to assess the generalizability of these findings. We lacked data on the latency from the attempted suicide to the plasma metabolomic assay. There is no benchmark for the optimal timeframe to assay metabolomics relative to suicide attempts. The characterization across time of the plasma metabolome following a suicide attempt may be addressed in future longitudinal studies. Metabolites were not collected in a systematic manner (e.g., under fasting conditions, at a uniform time of day), which may confer noise to the analyses, although fasting status may not significantly impact laboratory variability for most metabolites (Townsend et al., 2013). Other psychiatric metabolites, including dopamine and GABA, are not assayed by the p180 platform, and the platform was unable to detect serotonin at the quality control threshold employed. Lipids assayed by current mass spectrometry technology may actually reflect sum signals of all isomeric/isobaric compounds having the same parent and daughter ions (Sciences, 2020). Therefore, future studies should validate the identified lipid metabolites with additional assays.

Future studies may build on this work by examining whether biological variation in the top SNVs and metabolites can be used prospectively to predict suicide attempts. Those predictions may be bolstered by additional risk measures, such as assays of cerebrospinal fluid, brain imaging, and clinical history (Hayashi-Takagi et al., 2014). Neuroimaging studies suggest that complex interactions among ventral and dorsal prefrontal cortex systems produces a framework for suicidal ideation to progress to lethal behaviors (Schmaal et al., 2020). At present, however, distinct neural signatures with imaging are not sufficiently developed for clinical implementation (Mann and Rizk, 2020). Combinations of neuroimaging and peripheral biomarker measurements may yield more accurate biosignatures for suicidal behavior than either modality alone (Calati et al., 2020). Furthermore, given the circadian variants implicated in this work, future studies might assess the utility of peripheral sleep monitors in helping to predict suicidality (e.g., smartwatches). Sleep complaints and disorders are associated with suicide attempts (Bernert and Joiner, 2007; Mirsu-Paun et al., 2017; Benard et al., 2019; Mash et al., 2021). In our sample, QIDS-C measures of insomnia, hypersomnia, and energy/fatiguability did not significantly differentiate individuals with and without a history of suicide attempt in the current study, although sleep-onset insomnia and early-morning insomnia had marginal significance. These seemingly contradictory findings may be attributed to the discrepancy between self-reported sleep quality and sleep quality determined by wrist-worn actigraphy monitors in depressed patients (Orta et al., 2016). This calls for smartwatch sleep monitoring of depressed patients would provide richer, remote, and scalable data towards quantifying sleep patterns and sleep quality in individuals at risk of suicide. Biosignatures developed with such data could ultimately identify subgroups of patients with MDD who may benefit from care provided by specialty providers (e.g., psychiatrists specializing in suicidality). For example, biosignatures could inform on ideal candidates for the investigational circadian-based intervention for managing depressive symptoms, such as Circadian Reinforcement Therapy (CRT) (Dunker Svendsen et al., 2019). The network analysis suggests that biosignatures could also facilitate pharmacogenomic-guided antidepressant prescriptions aimed towards personalizing care.

In conclusion, this study utilized a network science approach to derive molecular signatures of lifetime history of suicide attempt and antidepressant response. The subnetworks of genes and metabolites which best differentiate individuals with MDD by their lifetime history of suicide attempt also associate with antidepressant pharmacotherapy response exclusively in individuals with no lifetime history of suicide attempt. The interplay of genomics, metabolomics, treatment response, and lifetime history of suicide attempts in MDD indicates that multi-modal data integration is a critical step towards the goal of clinically-actionable, objective biomarkers to a) monitor patients at risk for suicide attempts and b) individualize therapy for subgroups of patients with MDD. The results encourage future studies to assay a wider range of -omics measures (e.g., transcriptomics, proteomics, and epigenomics) and to include digital technologies (e.g., smartwatches) to augment understanding of the associations derived in this work. Therefore, this work represents a starting point for additional multi-modal studies aimed at improving our mechanistic understanding of suicidality and antidepressant response in patients with MDD.

## Data Availability

The original contributions presented in the study are included in the article/Supplementary Materials, further inquiries can be directed to the corresponding authors.
