# Supplementary material for: Network science approach elucidates integrative genomic-metabolomic signature of antidepressant response and lifetime history of attempted suicide in adults with major depressive disorder
Source: Front Pharmacol. 2022 Oct 3;13:984383. doi: 10.3389/fphar.2022.984383 (PMC9573988; doi:10.3389/fphar.2022.984383)
Supplement: Supplementary file 2 [file Table1.DOCX]

**Supplementary Materials**

**TITLE:** Network Science Approach Elucidates Integrative Genomic-Metabolomic Signature­­ of Antidepressant Response and Lifetime History of Attempted Suicide in Adults with Major Depression Disorder

**PREVIOUS PRESENTATION:** Poster presentation at the American College of Neuropsychopharmacology (2021) with title “Multi-Omic Characterizations of Suicide Attempts in Adults with Major Depressive Disorder Reveals Associations of Circadian Genes and Plasma Metabolites”

**AUTHORS:** Caroline W. Grant*, B.A.^1^, Angelina R. Wilton*^1,2^, Rima Kaddurah-Daouk, Ph.D.^3^, Michelle Skime, M.S.^4^, Joanna Biernacka, Ph.D.^5^, Taryn Mayes M.S.^6^, Thomas Carmody, Ph.D.^7^, Liewei Wang, M.D., Ph.D.^1^, Konstantinos Lazaridis, M.D.^8^, Richard Weinshilboum, M.D.^1^, William V. Bobo, M.D., M.P.H.^9^, Madhukar H. Trivedi M.D.^6^, Paul E. Croarkin, D.O, M.S^4*,^ and Arjun P. Athreya, Ph.D. ^1*,^

**AUTHOR AFFILIATIONS**

1. Department of Molecular Pharmacology and Experimental Therapeutics, Mayo Clinic, Rochester, MN, USA.

2. Department of Molecular and Integrative Physiology, University of Illinois at Urbana-Champaign, IL, USA

3. Department of Psychiatry and Behavioral Sciences, Department of Medicine, Duke Institute for Brain Sciences, Duke University, Durham, NC, USA.

4. Department of Psychiatry and Psychology, Mayo Clinic, Rochester, MN, USA.

5. Department of Quantitative Health Sciences, Mayo Clinic, Rochester, MN, USA.

6. Peter O’Donnell Jr. Brain Institute and the Department of Psychiatry at the University of Texas Southwestern Medical Center, Dallas, TX, USA.

7. Department Population and Data Sciences at the University of Texas Southwestern Medical Center in Dallas

8. Department of Internal Medicine, Division of Gastroenterology and Hepatology, Mayo Clinic, Rochester, MN, USA

9. Department of Psychiatry and Psychology, Mayo Clinic, Jacksonville, FL, USA.

*These authors contributed equally to this work.

**Corresponding author:**

Arjun P. Athreya, M.S., PhD.,

Dept. of Molecular Pharmacology and Exp. Therapeutics,

Mayo Clinic,

200 First St. SW,

Rochester, MN – 55902

Tel: +1-507-422-6073

Email: athreya.arjun@mayo.edu

**FIGURES**

**Figure Legends**

**Supplementary Figure SI.** Sample inclusion by study.

**TABLES**

**Table Legends**

**Supplementary Table SI.** Metabolites common to PGRN-AMPS and CO-MED and included in multi-omics integration analysis.

**Supplementary Table SII.** SNVs identified as correlates of suicide attempts in the PubMed and GWAS Catalog searches. Nearest gene annotations and alternate allele frequencies are according to the NCBI RefSeq Database. Study cohort clinical diagnosis describes the patient cohort of each publication where SNVs associated with suicide attempts. ORF: Open reading frame. SNV annotations are according to the dbSNP database.

**Supplementary Table SIII.** Associations of SNVs with suicide attempt history. All tests had 2 degrees of freedom. For descriptive purposes, SNVs are labelled by rsID and nearest gene annotated by the NCBI RefSeq Database.

**Supplementary Table SIV.** Pearson correlation coefficients for significant associations (|r| > 0.1, p < 0.05) between SNVs and metabolites in either network (suicide attempters or non-attempters). For descriptive purposes, SNVs are labelled by rsID and nearest gene annotated by the NCBI RefSeq Database.

**Supplementary Table SV.** Population frequencies for SNVs included in the multi-omics integration network analysis according to the gnomAD v2.1.1 browser (https://gnomad.broadinstitute.org). For descriptive purposes, SNVs are labelled by rsID and nearest gene annotated by the NCBI RefSeq Database.

**Supplementary Table SVI:** QIDS-C items association with lifetime attempted suicide by Kruskal-Wallis test.

**Supplementary Figure I: Sample inclusion by study**

Yes

Yes

No

No

**Multi-Omics Set**

PGRN-AMPS: 245

CO-MED: 103

No

PGRN-AMPS: 19

CO-MED: 8

Genotyping?

Suicide Attempt History Available?

PGRN-AMPS: 0

CO-MED: 47

No

Yes

PGRN-AMPS: 0

CO-MED: 1

< 20% individual data missing?

Yes

PGRN-AMPS: 529

CO-MED: 665

Baseline metabolomics?

PGRN-AMPS: 265

CO-MED: 506
